# Rutacecarpine Inhibits Angiogenesis by Targeting the VEGFR2 and VEGFR2-Mediated Akt/mTOR/p70s6k Signaling Pathway

**DOI:** 10.3390/molecules23082047

**Published:** 2018-08-15

**Authors:** Lijun Ji, Mingfei Wu, Zeng Li

**Affiliations:** 1 School of Pharmacy, Anhui Medical University, Hefei 230032, China; lkzh1020@163.com (L.J.); 18956063580@163.com (M.W.); 2 School of Basic Medicine, Anhui Medical University, Hefei 230032, China

**Keywords:** Ru, angiogenesis, reverse screen, VEGFR2

## Abstract

This study aimed to investigate the effect of Ru (Rut) on angiogenesis, and the underlying regulation mechanism of signal transduction. 3-(4,5-dimethyl-2-thiazolyl)-2,5-diphenyl-2*H*-tetrazolium bromide (MTT) assay, adhesion inhibition experiment, migration inhibition experiment, and chick embryo chorioallantoic membrane (CAM) assays were performed on models of angiogenesis. The potential targets of rutaecarpine (Ru) were reverse screened with Discovery Studio 2017. The interaction between the compound and target were detected by surface plasmon resonance (SPR), enzyme-activity experiment, and Western blot assay. The obtained results confirmed that Ru exhibited modest inhibitory activity against human umbilical vein endothelial cells (HUVECs) (IC_50_ =16.54 ± 2.4 μM) and remarkable inhibitive effect against the migration and adhesion of HUVECs, as well as significant anti-angiogenesis activities in the CAM assay. The possible targets of vascular endothelial growth factor receptor 2 (VEGFR2) were identified by computer-aided simulation. Results showed a good binding relationship between the ligand and target through molecular docking, and this relationship was confirmed by SPR analysis. Furthermore, enzyme-activity experiment and western blot assay showed that Ru remarkably inhibited the activity of VEGFR2 and blocked the VEGFR2-mediated Akt/ (mTOR)/p70s6k signaling pathway in vitro. Ru can be a potential drug candidate for cancer prevention and cancer therapy.

## 1. Introduction

Malignant tumors are some of the major diseases that seriously affect human health and threaten human life. Malignant tumors have become the second highest cause of human death and they are difficult to treat [1]. Angiogenesis is a key process in tumor growth and metastasis, and the largest tumor can grow to 1–2 mm^3^ without angiogenesis. The use of traditional chemotherapy drugs is easy [2,3]. Accordingly, a new strategy for cancer treatment that inhibits the growth of tumors by blocking the generation of tumor neovascularization has been developed [4]. Endothelial cells, which are found in the inner lining of blood vessels, compose the fundamental portion of new and pre-existing blood vessels. A complete process of angiogenesis involves the proliferation, migration, and differentiation of endothelial cells. Thus, a large number of discovered angiogenesis inhibitors target endothelial cells [5,6,7]. In pathological and physiological angiogenesis, vascular endothelial growth factor (VEGF) and its receptors (VEGFRs) play crucial roles. The signaling cascade mediated by VEGF/VEGFR2 regulates the proliferation, migration, survival, and permeability of vascular endothelial cells. Considering the importance of VEGFR2 in angiogenesis, this receptor is the vital target in anti-angiogenic therapy against cancer [8,9,10].

Ru is a major component of *Evodia rutaecarpa*, and it is widely used in traditional medicine to treat many diseases. Ru has anti-tumor potential by inhibiting cell proliferation and inducing apoptosis via various molecular mechanisms [11,12,13,14]. In this research, Ru showed a remarkable inhibitive effect against the migration and adhesion of human umbilical vein endothelial cells (HUVECs), and significant anti-angiogenesis activities in the chorioallantoic membrane (CAM) assay. However, the anti-angiogenic mechanisms of Ru have not been reported.

A large number of protein structures have been resolved, with advances in X-ray crystal diffraction and nuclear magnetic resonance techniques. Specifically, the group that contributes to the inhibitor activity and its spatial distribution can be obtained from the complex structure when the structures of the receptor and inhibitor complexes are known. Thus, computer-aided simulation on the construction of a pharmacophore model based on the function of the active ligand-building pharmacophore model and receptor-ligand crystal complex can remarkably shorten experiments. [15,16]. On this basis, we used reverse virtual screening to precisely search the target of Ru as an angiogenesis inhibitor. We identified the possible targets for VEGFR2 by computer-aided simulation, and we found a good binding relationship between Ru and VEGFR2 through molecular docking and molecular dynamics simulation. Surface plasmon resonance (SPR) strengthened the evidence that links Ru with VEGFR2. Furthermore, we identified one mechanism of action involving the inhibition of VEGFR2 and the blockage of the VEGFR2-mediated Akt/mammalian target of rapamycin (mTOR)/p70s6k signaling pathway.

## 2. Results

### 2.1. Cytotoxicity Against HUVECs

Endothelial cells, which come from the inner lining of blood vessels, make up a fundamental portion of new blood vessels, as well as pre-existing ones. A complete angiogenesis process includes the proliferation, migration, and differentiation of endothelial cells, so a great deal of angiogenesis inhibitors that have been discovered to date, target endothelial cells.

**Figure 1:**
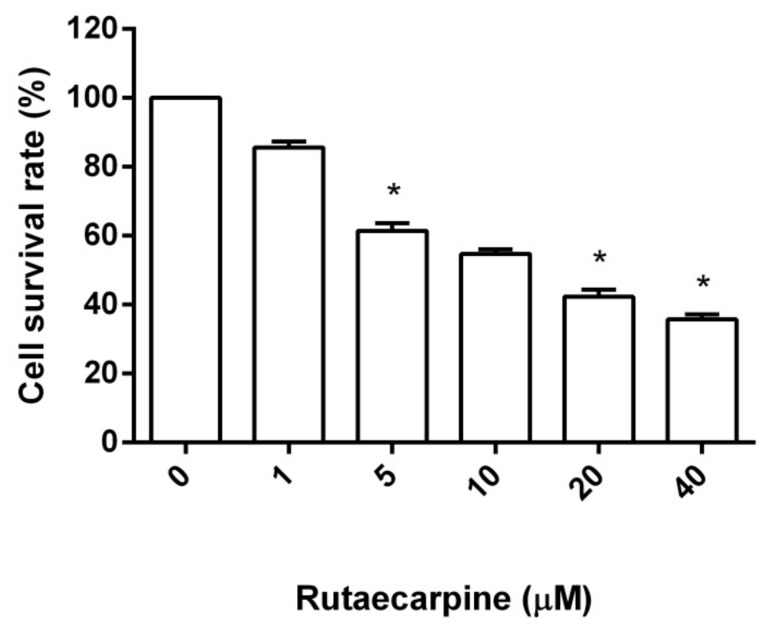
Cytotoxicity of Ru on human umbilical vein endothelial cells (HUVECs) measured by MTT assay, * *p* < 0.05 versus 0 µM control. All measurements were obtained in triplicate.

Cell viability was determined by 3-(4,5-dimethyl-2-thiazolyl)-2,5-diphenyl-2*H*-tetrazolium bromide (MTT) assay. The cells were treated with different concentrations of Ru (0, 1, 5, 10, 20, and 40 μM) for 48 h. MTT colorimetric results showed that the proliferation inhibition rate of HUVEC cells increased with increased Ru concentration (Figure 1). At the same time, the proliferation inhibition rate of Ru-treated HUVEC cells increased, and the difference was significant (*p* < 0.05) compared with that of the control group. Ru exhibited modest inhibitory activity against HUVECs, with an IC_50_ of 16.54 ± 2.4 μM by using a fitting formula.

### 2.2. Inhibition of the Adhesion of HUVECs

**Figure 2:**
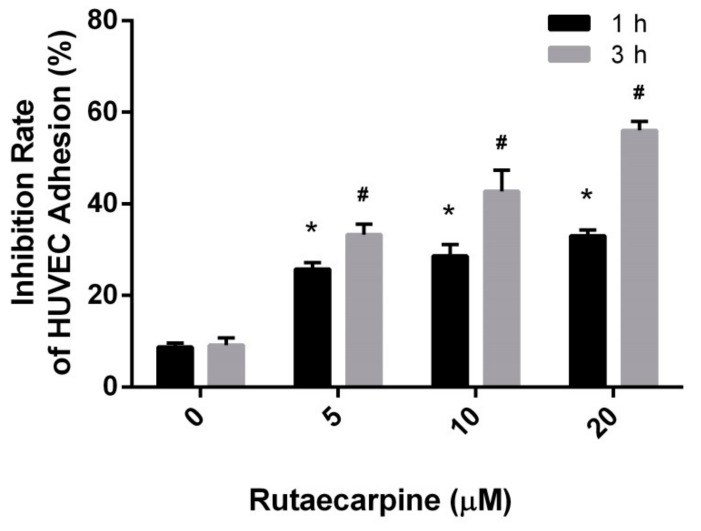
Inhibitory effects of Ru on the adhesion of HUVECs in collagen for 1 h (* *p* < 0.05 versus 0 µM control) and 3 h (^#^ *p* < 0.05 versus 0 µM control) at doses of 5, 10, and 20 μM. Independent experiments were performed throughout the in vitro studies in triplicate.

A perfect angiogenesis inhibitor must show modest toxicity toward HUVECs and high inhibitive capability for the proliferation, migration, and differentiation of HUVECs. Three subsequent assays, namely, HUVEC adhesion, HUVEC migration, and CAM assays were adopted to assess the inhibitory effects of Ru. Cell adhesion assays were conducted to assess the inhibitory effects of Ru on the attachment of endothelial cells to type I collagen. As shown in Figure 2, Ru remarkably inhibited the adhesion of HUVECs at 1 h and 3 h. The inhibition rate increased due to Ru exposure in a concentration-dependent manner. The inhibitory rates (1 h) were 25.8 ± 1.4%, 28.6 ± 2.5%, and 33.1 ± 1.3% and the inhibition rates (3 h) were 33.3 ± 2.0%, 42 ± 3.8% and 56.1 ± 2.1% when the compound concentrations were 5, 10, and 20 μM, respectively.

### 2.3. Inhibition on the Migration of HUVECs

**Figure 3:**
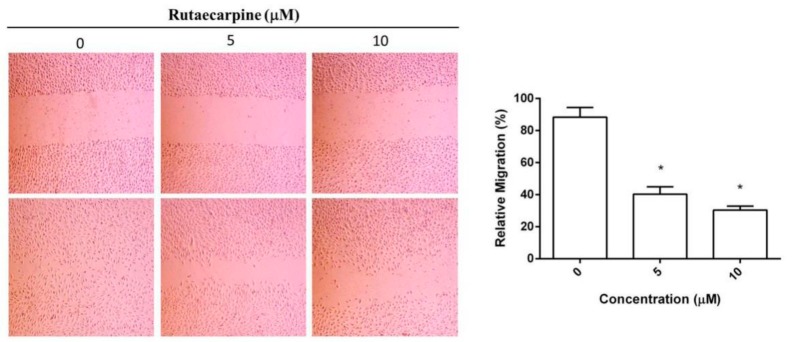
HUVECs are treated with Ru for 24 h, and migration (scale bar, 200 µm) is determined by wound healing assay. Independent experiments were performed throughout the in vitro studies in triplicate. * *p* < 0.05 versus 0 µM control.

The migration of HUVECs is an essential step in angiogenesis. The effect of Ru on the chemotactic motility of HUVECs is shown in Figure 3. The migration distances and invasive cell numbers remarkably decreased in a dose-dependent manner upon treatment of HUVECs with 5 μM or 10 μM Ru for 24 h. Compared with the control group, the migration rates decreased to 40.2 ± 4.6% and 31.7 ± 3.1% (*p* < 0.05). This finding indicated that Ru had an inhibitory effect on the migration of endothelial cells, thereby supporting the results of adhesion studies.

### 2.4. Ru Inhibited Angiogenesis In Vivo

**Figure 4:**
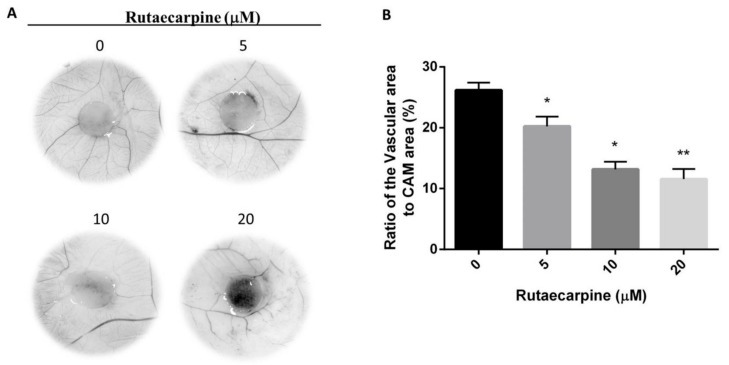
Results of CAM assay (**A**). NaCl solution (0.9%) is used as a negative control. Image-Pro Plus 5.0 was used to calculate the vascular area and CAM area (**B**). Ratio of the vascular area to CAM area showed a significant difference between the Ru-treated groups and control group, * *p* < 0.05, ** *p* < 0.01. Independent experiments were performed in triplicate.

A CAM assay was conducted to assess the effect of Ru on anti-angiogenesis activities in vivo. As shown in Figure 4, a remarkable reduction was observed on the angiogenic responses when various concentrations of Ru were added on CAMs. Ru at tested concentrations of 10 μM inhibited a noticeable degree of proliferation of new blood vessels, which was remarkably higher than that of the normal group. Among the four concentrations, 20 μM concentration exhibited the highest anti-angiogenic activity with inhibition of neovascularization.

### 2.5. Target Prediction

**Figure 5:**
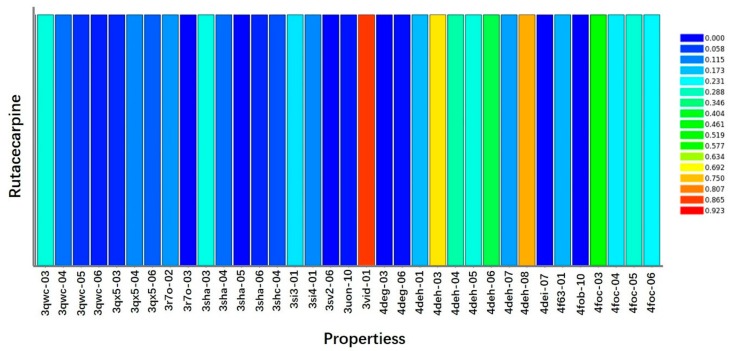
Profiling of the predicted protein targets of Ru via DS 2017. The y-axis represents the compound Ru, and the x-axis indicates the predicted pharmacophore models (pharmacological targets) of Ru. The color from blue to red represents a high fit value and a better fit.

The molecular targets of Ru were predicted by using Discovery Studio 2017 (DS 2017) software (Figure 5). The fit value indicated the score of the ligand binding to the receptor, and a higher value meant better binding. The ranking based on fit score was arranged in descending order, and the top 10 disease-related targets are shown in Table 1. Among these targets, VEGFR2 was focused, which was a critical target-related tumor angiogenesis.

### 2.6. Ru Directly Bound to VEGFR2 by Molecular Docking and SPR Assay

**Figure 6:**
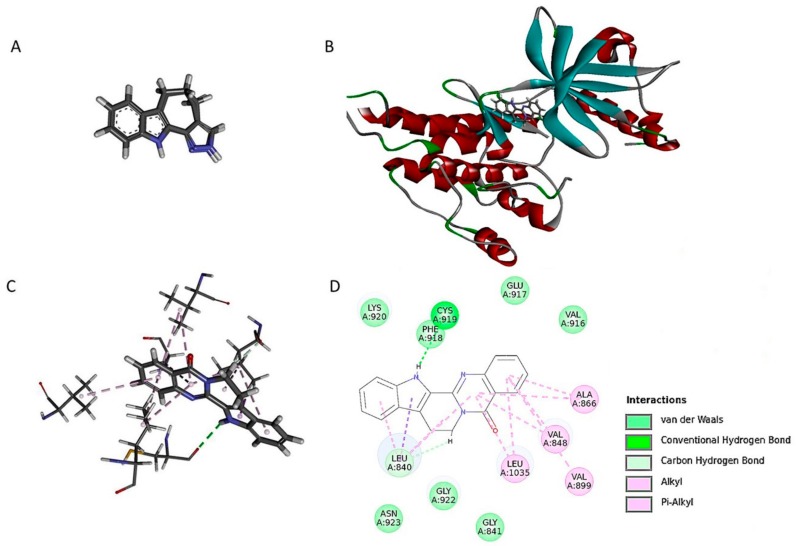
Binding mode of Ru with VEGFR2 (PDB code: 3VID). Docking the original ligand (**A**). (**B**). 3D diagram of Ru inserted in the VEGFR2 binding site. (**C**). For clarity, only interacting residues are displayed. (**D**). 2D diagram of the interaction between Ru and amino acid residues of the nearby active site.

Molecular docking experiments were performed to understand the binding mode between the ligand and target. A re-docking protocol was performed on co-crystallized structure of VEGFR2 (PDB entry: 3jpv) in the docking study. On each re-docked pose, the competency assessment was evaluated by considering the root-mean-square deviation (RMSD) values. The model was proved to be reliable (RMSD: 0.5417A). These results suggested that the LibDocK protocol was able to produce the convincing re-docking results for cognate ligand within the binding pocket of VEGFR2. The docking results showed that the active site was combined with Ru to produce 15 postures. We determined that 92.3 kcal/mol was the docking energy value of the highest scoring position in the docking results (as evaluated by LibDockScore). As shown in the binding model (Figure 6), Ru was nicely bound to the active site of VEGFR2 by one hydrogen bond, with CYS A: 919 and one pi-sigma bond with LEU A:804. Furthermore, other weak interactions, such as pi-alkyl, alkyl, and carbon-hydrogen bonds, contributed to the binding affinity of Rut with VEGFR2.

**Figure 7:**
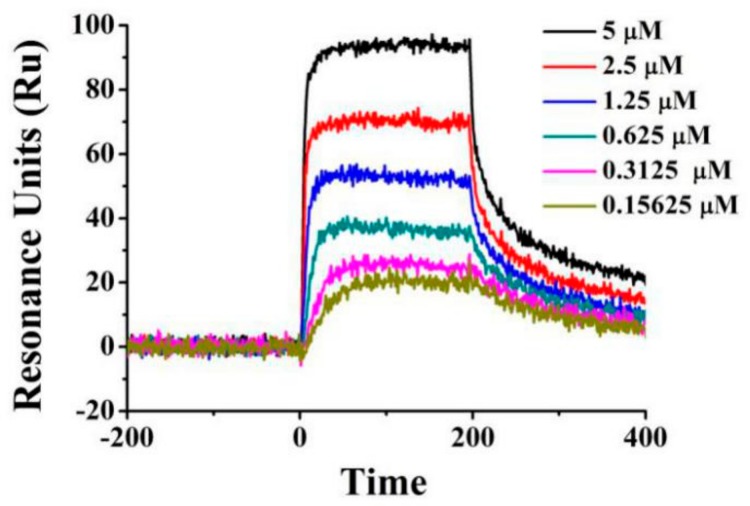
Surface plasmon resonance (SPR) sensograms for Ru binding to the immobilized VEGFR2. As shown in the plot, ligand concentrations in the flow solutions were 5, 2.5, 1.25, 0.625, 0.3125, and 0.15625 μM for the curves from bottom to top.

To confirm the interaction of Ru with VEGFR2, an SPR-based Biacore T200 biosensor was used to measure the binding affinity of Ru with VEGFR2. VEGFR2 protein was immobilized on the sensor chip, and binding responses in response units (RUs) were continuously recorded and graphically presented as a function of time in sensograms. The association of Ru with VEGFR2 was evaluated using the equilibrium dissociation constant (KD) by fitting the sensogram with a 1:1 (Langmuir) binding fit model. As shown in Figure 7, Ru had a high binding affinity toward VEGFR2 in a concentration-dependent manner. KD was calculated to be 0.4706 μM. The combinations of molecular modeling studies and SPR results indicated that Ru may be a potential VEGFR2 ligand.

### 2.7. Ru Attenuates VEGFR2 Tyrosine Kinase Activity

**Figure 8:**
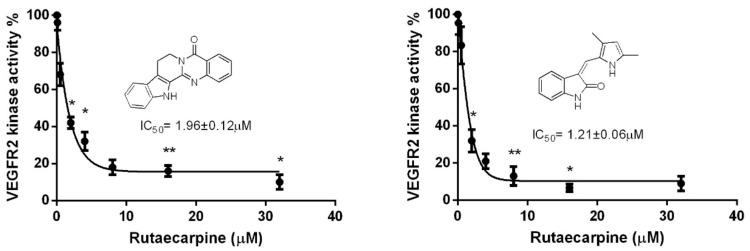
Inhibition of VEGFR2 kinase activity by Ru, and semaxanib is analyzed by using an in vitro HTScan^®^ VEGF receptor 2 kinase kit. Independent experiments were performed throughout the in vitro studies in triplicate. * *p* < 0.05, ** *p* < 0.01 compared to the control.

Previous studies indicated that the blockage of VEGFR2 activity can remarkably limit tumor neo-angiogenesis, and in VEGF-dependent angiogenesis, VEGFR2 plays a crucial role. So we investigated the inhibition on the kinase activity of VEGFR2 by Ru, and the previously reported VEGFR2 receptor semaxanib was used as a positive control [17]. Thus, to evaluate the effects of Ru on VEGF-stimulated P-VEGFR2, an enzyme linked immunosorbent assay (ELISA)-based tyrosine kinase assay was conducted. Ru can dose-dependently suppress the kinase activity of VEGFR2 with an IC_50_ of 1.96 ± 0.12 μM, which is in the same order of magnitude as the reference compound semaxanib (IC_50_ = 1.21 ± 0.06 μM) (Figure 8).

### 2.8. Ru Inhibits the Activation of VEGFR2-Mediated Akt/mTOR/p70S6K Signaling in HUVECs

**Figure 9:**
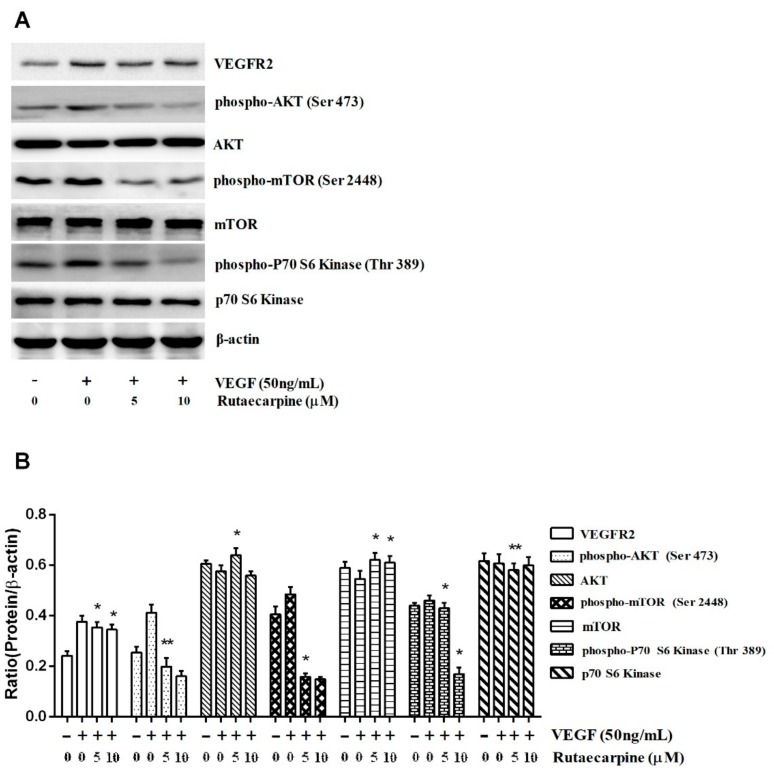
Ru inhibited the activation of AKT/mTOR/P70S6K signaling in HUVECs. Western blot analysis (**A**) and quantitative data of protein (**B**) Independent experiments were performed throughout the in vitro studies in triplicate. * *p* < 0.05, ** *p* < 0.01 compared to the control.

Ru remarkably suppressed the activation of VEGFR2 downstream signaling molecules, such as Akt, mTOR, and p70S6K (Figure 9), which indicated that Ru inhibited angiogenesis on the surface of HUVECs through the direct inhibition of VEGFR2. Extensive downregulation of phospho-Akt (Ser 473), which is a well-known downstream target of VEGFR2, was observed at Ru. However, the total Akt levels remained unchanged. Next, we assessed the expression of phospho-mTOR (Ser 2448) and phospho-p70S6K (Thr 389) after Ru exposure. The results in Figure 9 revealed that phospho-mTOR and phospho-p70S6K levels also increased with phospho-Akt, and the total mTOR and p70S6K levels were unaltered. We suggested that Ru inhibited tumor angiogenesis by blocking the Akt/mTOR/p70S6K signaling pathway. Our result demonstrated that Ru exerted its anti-angiogenic effect by selectively targeting certain signaling events at VEGFR2 downstream.

## 3. Materials and Methods

### 3.1. Materials

Ru was purchased from Sigma (St. Louis, MO, USA) and was dissolved in 100% dimethyl sulfoxide (DMSO). A stock solution of 10 mmol/L Ru was prepared and stored as small aliquots at −20 °C for future use. MTT, DMSO, and rat tail collagen type I were purchased from Sigma-Aldrich Co (St. Louis, MO, USA). The primary polyclonal rabbit antibodies (anti-PI3K, anti-AkT, anti-mTOR, and anti-p70S6K) and horseradish peroxidase (HRP)-conjugated anti-rabbit antibodies were purchased from Elabscience Biotechnology Co., Ltd. (Shanghai, China). Antibodies against β-actin, phospho-specific antibodies (anti-Akt (Ser 473), anti-mTOR (Ser 2448), and anti-p70S6K (Thr 389)) were purchased from Cell Signaling Technology (Danvers, MA, USA). TRIzol reagent, protease inhibitor cocktail, polyvinylidene difluoride (PVDF) membranes, and sodium dodecyl sulfate polyacrylamide electrophoresis (SDS–PAGE) gels were acquired from Beyotime Biotechnology (Haimen, China). Phosphate-buffered saline (PBS), Dulbecco’s Modified Eagle’s Medium (DMEM), and fetal bovine serum (FBS) were obtained from Thermo Fisher Scientific (Waltham, MA, USA). VEGFR2 protein and its ELISA test kits were purchased from Cell Signaling Technology (Danvers, MA, USA).

### 3.2. Cell Line and Cell Culture

HUVEC lines were obtained from the Anhui Medical University College of Pharmacy, Anhui Province, China. HUVECs were cultured in DMEM (HyClone, Logan, UT, USA) supplemented with 10% FBS (HyClone, Logan, UT, USA) and streptomycin/penicillin (100 U/mL). Cells were cultured at 37 °C humidified atmosphere with 5% CO_2_.

### 3.3. Cell Viability Assay

HUVEC viability was assessed by the MTT assay. The cells (5 × 10^3^ cells/well) were seeded in a 96-well plate with DMEM medium supplemented with 10% FBS. The culture medium was removed, and the cells were rinsed twice with PBS after the cells were allowed to adhere. HUVECs (5 × 10^3^ cells/well) were treated with fresh medium supplemented with 10% FBS and various concentrations of Ru (0, 1, 5, 10, 20, and 40 μM) at 37 °C for 48 h. After incubation, MTT solution (5 mg/mL) was added, and the plate was incubated for an additional 4 h. DMSO (100 μL) was added in each well, the resulting formazan deposit was solubilized in DMSO, and the optical density (OD) was recorded at 490 nm. All measurements were obtained in triplicate.

### 3.4. Extracellular Matrix Adhesion Assay

Rat tail collagen type I (5 mg/mL) was diluted to 0.012 mg/mL with sterile acetic acid solution (0.006 mol/mL). Rat tail collagen type I solution (containing 2 μg collagen) was added to a 96-well cell culture plate at 50 μL per well, and the blank wells were set (without collagen). The 96-well plate was left to dry on a clean bench overnight. After washing three times with PBS, an FBS solution in PBS (0.2%) was added to each well and the 96-well plate was incubated at 37 °C for 2 h. After culturing, the 96-well culture plate was washed three times with PBS. HUVECs (2 × 10^5^ cells/well) were added to the corresponding wells at 50 μL per well, and the medium that contained different concentrations of the compound was added to the corresponding wells at 50 μL per well. The control wells (compound-free medium 50 μL) were set, and the plate was placed in the incubator and was cultured for 1 h and 3 h. After incubation, the plates were washed three times with PBS to remove non-adherent cells. Adherent cells were stained with 0.2% crystal violet in 20% methanol for 10 min, and the staining solution was discarded, rinsed, and air-dried. SDS solution (2%) was added to the wells of the plate, and absorbance OD was read at 570 nm on a microplate reader. Each hole was tested in parallel three times.

### 3.5. Wound Healing Migration Assay

Wound healing migration assay was performed by plating cells in logarithmic phase in 6-well culture dishes. Monolayer HUVECs were scratched along a straight line using a 200 μL pipette tip in each group, and the scraped cells and cell debris were washed with PBS for three times. Subsequently, the scratched monolayers were cultured in fresh serum free medium with various drug concentrations (0, 5, and 10 μM) at 37 °C for 24 h. Then, the migrating cells were captured with an inverted microscope at 100 × magnification. Images were captured at 0 h and 24 h at the same location of scratch. The migrated cells were observed from three randomly selected fields and were quantified by manual counting. The migration rate was the ratio of the migrated distance to the initial distance. Inhibition percentage was expressed as the percentage of untreated cells (100%). The assay was repeated three times independently.

### 3.6. Chick CAM Assay

CAM assay was performed to determine the in vivo anti-angiogenic activity of Ru. Briefly, fertilized chick embryos were pre-incubated at 37 °C in 60–80% humidity for six days. Eggs were cleaned with a 70% alcohol solution, in which a hole was drilled through the pointed pole of the shell and saline injection was dropped in the egg shell membrane. The egg shell membrane was aseptically removed. Part of the CAM of the embryo was exposed by peeling a 1.5–2.0 cm window in the shell, which exposed the underlying blood vessels. Different concentrations of drug sensitive paper were placed on the CAM. The window was sealed with clear adhesive tape, and eggs were re-subjected for incubation. A minimum of eight eggs were used per group. After the drug sensitive paper implantation, host eggs were incubated for another 48 h. The eggs were transferred to a 4° refrigerator for 24 h. At day 10 of embryonic development, eggs were collected from the refrigerator and the CAM was cut with scissors (2–3 cm in diameter) centering on the drug sensitive paper. The implants and surrounding embryonal tissues were surgically removed and fixed in 10% formaldehyde. A blind, independent observer analyzed the CAM response. Image-Pro Plus 5.0 (Bethesda, MD, USA) was used to calculate the vascular area and CAM area.

### 3.7. Molecular Modeling

To understand the potential interactions between the tested drug and selected protein, reverse direction finding were performed using DS 2017 R2 in this study. The ligand structure was drawn by using the ChemDraw program. Ligand structures were optimized by using DS. Protein and ligand were prepared for the reverse target and docking simulation by adding partial charges and hydrogen with the aid of DS. The proteins can be acquired from the database of Protein Data Bank (PharmaDB pharmacophores, New York, NY, USA). Ligand and pharmacophores were matched in DS.

The X-ray crystal structure of VEGFR2 complexed with the inhibitor was obtained from the RCSB Protein Data Bank (PDB code:3VID, New York, NY, USA). The crystal structure of VEGFR2 (3VID) contains the native ligand. Vina docking encoded in DS 2017 R2 software (Beijing, China) was employed to identify the potential binding of Ru to VEGFR2. The binding pocket was defined by the center of native ligand 4TT. Docking parameters were set to default values. All docked poses of Ru were clustered using a tolerance of 2 A for RMSD and were ranked on the basis of the binding docking energies. The lowest energy conformation in the most populated cluster was selected for subsequent study.

### 3.8. SPR Assay

SPR37 is used as a functional assay to demonstrate the interaction capability between Ru and VEGFR2. SPR experiments were performed at 25 °C with a Biacore T200 apparatus on CM5 sensor chips (GE Healthcare, Fairfield, CT, USA). CM5 sensor chip was activated, and VEGFR2 protein in 10 mM NaAc (pH 5.5) was immobilized at densities of approximately 400 RUs. The samples were prepared in PBS with Tween 20 (PBS-T) (137 mM NaCl, 2.7 mM KCl, 1.5 mM KH2PO4, 8.1 mM Na2HPO4, 0.05% Tween 20, pH 7.35) running buffer and were injected over the functionalized surface at a flow rate of 30 µL/min for an association phase of 120 s and a dissociation phase of 90 s. After each injection, the sensor chip surface was completely regenerated with PBS (pH 7.4) at a flow rate of 10 µL/min for 60 s. Running buffer (blank control) was made to flow over the chip, and sensorgrams were obtained after blank subtraction. The sensorgrams were analyzed with the Biacore T200 evaluation software (version 2.0, Fairfield, CT, USA). The kinetic parameters, including association rate constants (ka), dissociation rate constants (kd), and kinetic dissociation constant (KD), were calculated by Biacore T200 evaluation software 2.1 (Fairfield, CT, USA).

### 3.9. Western Blotting

HUVECs in logarithmic phase were treated with various concentrations of the compound (0, 5, and 10 μM) for 48 h. The samples of total cellular protein extracts were loaded and separated by SDS-PAGE and were transferred on PVDF membranes (Beyotime Biotechnology, Haimen, China). The membranes were blocked with 5% dehydrated skim milk in TBST for 2 h at room temperature. The blots were washed thrice in TBST buffer and were incubated overnight at 4 °C with primary antibodies against β-actin (1:1000 dilution), Akt (1:1000 dilution), mTOR (1:1000 dilution), and p70S6K (1:1000 dilution) (all from Elabscience Biotechnology Co., Ltd., Shanghai, China), and p-Akt (Ser 473, 1:2000 dilution), p-mTOR (Ser 2448, 1:1000dilution), and p-p70S6K (Thr 389, 1:1000dilution) (all from Cell Signaling Technology, Inc., Danvers, MA, USA). The blots were washed thrice in TBST buffer, followed by the addition of secondary antibodies. The unbound antibodies in each step were washed with TBST three times. The specific proteins in the blots were visualized using an enhanced chemiluminescence reagent (Thermo Scientific, Waltham, MA, USA). Immunoreactivity of the membranes were detected using the Bio-Rad-ImageLab with an electrochemiluminescence system (Thermo Fisher Scientific, Waltham, MA, USA). The densitometry of the protein bands was measured using the ImageJ (NIH image software) and was normalized to their relevant controls.

### 3.10. Statistical Analysis

Results are expressed as mean ± standard deviation. Statistical comparisons were performed using Student’s *t*-test and one-way ANOVA. The minimum level of significance was *p* < 0.05.

## 4. Discussion and Conclusions

Angiogenesis, which is the maintenance and formation of blood vessel structures, is essential for the physiological functions of tissues and angiogenesis, which is the maintenance and formation of blood vessel structures, is important for the progression of diseases such as cancer and inflammation [18,19]. In recent decades, various signaling molecules, such as VEGF-VEGFRs, have been identified to play important roles in angiogenesis. VEGFR2 is expressed on the surface of most blood endothelial cells. VEGF, which is also known as VEGF-A, is a protein with vascular permeability activity [20]. VEGF plays an important role in the proliferation and migration of vascular endothelial cells [21]. The existing research shows that many endothelial cells caused by VEGF physiological or pathological changes are mainly mediated by VEGFR2. These adjustments include proliferation, migration, survival, and permeability changes [22]. Phytochemical-mediated anti-angiogenic intervention has attracted significant attention that is suitable as an effective cancer prevention strategy. Ru, which belongs to the quinazolinocarboline alkaloid class, is the main bioactive component isolated from *Evodiae fructus* [23]. Previous studies showed that Ru has many biological activities, such as anti-inflammatory, anti-obesity, anti-hypertensive, and it is used for the treatment of cardiovascular diseases [24,25,26,27,28]. The anti-tumor activity of Ru is weak, and its underlying mechanism is unclear [29]. However, the anti-angiogenic effects of Ru have not been reported.

Our study demonstrated that Ru played a remarkable role in inhibiting angiogenesis. Ru exhibited modest inhibitory activity with an IC_50_ value of 16.54 ± 2.4 μM. Ru remarkably inhibited the migration in endothelial cells and suppressed the adhesion of HUVECs. Furthermore, we evaluated the anti-angiogenic efficacy of Ru by using the chick CAM assay. Ru remarkably inhibited the VEGF-induced neovascularization in the chick CAM assay. Our present study shows that Ru is a potential inhibitor of angiogenesis in vitro.

The molecular targets of Ru were predicted using DS software. Ru bound well with VEGFR2 receptor through computer-aided simulation. SPR strengthened the evidence. In the present study, Ru remarkably blocks the kinase activity of VEGFR2 via downregulation of VEGF-induced phosphorylation of VEGFR2 expression as observed by VEGFR2 kinase inhibition assay in vitro, which suggests that Ru is a potent VEGFR2 inhibitor. These results suggest that the anti-angiogenic effects of Ru are partially mediated by the inhibition of VEGR2 activation. Akt, a known serine/threonine kinase, plays the central role in a range of cellular functions, including cell growth, proliferation, migration, protein synthesis, and angiogenesis [30,31]. mTOR is a remarkable regulator of tumor growth, metastasis, and angiogenesis [32,33]. p70S6K kinase (p70S6K), which is a downstream of Akt, plays an important role in regulating tumor microenvironment and angiogenesis [34]. In addition, the Akt/mTOR/p70S6K signaling is recently identified as a novel, functional mediator in angiogenesis [35]. Ru treatment showed a sharp decrease in the phosphorylation of mTOR and p70S6K, and its upstream kinase, Akt, suggested that Ru suppresses tumor angiogenesis by inhibiting VEGFR2 and blocking its multiple downstream signaling components.

In summary, these results clearly demonstrated that Ru can be utilized as an anti-cancer drug by blocking the VEGF signaling pathways in HUVECs that lead to inhibition of neovessel growth. Ru inhibited angiogenesis growth by targeting the VEGFR2-mediated Akt/mTOR/p70S6K signaling pathway. Ru can be a potential drug candidate for cancer prevention and cancer therapy.

## Figures and Tables

**Table 1:** Top 10 putative protein targets of Ru predicted using Discovery Studio 2017.

Rank	PDB ID	Putative Target	Fit Value
1	3jpv-06-s	Proto-oncogene serine/threonine-protein kinase Pim-1	0.91901
2	2ivv-06-s	Proto-oncogene tyrosine-protein kinase receptor RET precursor	0.883419
3	3geq-05-s	Proto-oncogene tyrosine-protein kinase Src	0.876451
4	3vid-01-s	VEGFR2	0.868609
5	3ad4-01-s	Proto-oncogene tyrosine-protein kinase LCK	0.847169
6	3ad4-02-s	Proto-oncogene tyrosine-protein kinase LCK	0.844494
7	3p0n-06-s	Tankyrase-2	0.825883
8	3qru-04-s	Cyclin-dependent kinase 2	0.824893
9	3hrr-03-s	Aflatoxin biosynthesis polyketide synthase	0.82355
10	3tiz-04-s	Cyclin-dependent kinase 2	0.802779
